# Adhérence des prestataires du secteur privé à la politique de prise en charge des cas de paludisme simple à Madagascar

**DOI:** 10.11604/pamj.2019.32.79.14721

**Published:** 2019-02-14

**Authors:** Fidiniaina Mamy Randriatsarafara, Vatsiharizandry Mandrosovololona, Jean Claude Andrianirinarison, Antsa Nomenjanahary Rakotondrandriana, Armand Eugene Randrianarivo-Solofoniaina, Arsène Ratsimbasoa, Jean de Dieu Marie Rakotomanga

**Affiliations:** 1 Département de Santé Publique, Faculté de Médecine d’Antananarivo, Madagascar; 2 Direction de Lutte contre le Paludisme, Ministère de la Santé Publique, Madagascar; 3 Institut National de Santé Publique et Communautaire-Befelatanana, Antananarivo, Madagascar

**Keywords:** Connaissances, adhérence, paludisme simple, secteur privé, Knowledge, adherence, uncomplicated malaria, private sector

## Abstract

**Introduction:**

Cette étude se propose d’évaluer l’adhérence des professionnels de santé du secteur privé à l'utilisation du TDR-palu (Test de Diagnostic Rapide-palu) et à la prescription de l’ACT (Artemisinin-based combination therapy) en cas de paludisme simple.

**Méthodes:**

Une approche évaluative rétrospective et transversale a été menée en septembre et octobre 2015 auprès de 11 districts sanitaires repartis dans les quatre faciès épidémiologiques existant à Madagascar. Au total, 43 prestataires de soins issus de 39 formations sanitaires privées (FSP) ont été interviewés et visités.

**Résultats:**

Les prestataires déclarent avoir lu le manuel de prise en charge du paludisme dans 16,3% des cas (4/43). Seul le quart (25,6%) des prestataires dispose de TDR dans leur bureau. L’ACT a été cité par 83,7% des prestataires comme médicament de première intention pour traiter le paludisme simple. Dans la pratique, 55,6% des prestataires émettent des doutes sur les résultats des TDR. L’utilisation des antipaludéens malgré les résultats négatifs des TDR (38,2%) est plus fréquente chez ceux ayant émis des doutes (p = 0,03). Inversement, malgré un TDR positif, la moitié des prestataires ne prescrit pas d’ACT (50%). La non-participation aux revues périodiques du District sanitaire (p = 0,05) influence négativement l’adhérence aux politiques.

**Conclusion:**

La faible adhérence des prestataires de soin du secteur privé aux directives nationales sur la prise en charge des cas de paludisme simple interpelle sur l’intérêt d’encadrer davantage ce secteur.

## Abstract (en)

**Introduction:**

This study aims to assess the adherence of private health providers to the use of malaria rapid diagnostic tests (RDTs) and to the prescription of artemisinin-containing combinations (ACT) in patients with uncomplicated malaria.

**Methods:**

We conducted an analytical, retrospective and cross-sectional study in 11 Madagascar’s health districts divided into four epidemiological strata in September and in October 2015. A total of 43 health providers from 39 private health care facilities (PHF) were interviewed and visited.

**Results:**

Health providers declared having read the malaria management manual in 16.3% of cases (4/43). Only one quarter (25.6%) of health providers had RDTs in their office. ACT was reported as “first-line drug” for the treatment of uncomplicated malaria by 83.7% of health providers. In practice, 55.6% of health providers had doubts about the results of the RDTs. The use of antimalarial drugs, despite having had negative RDTs results (38.2%), was more frequent among those who had raised doubts (p = 0.03). Conversely, despite having had positive RDTs results, half of the health providers did not prescribe ACT (50%). The decision to not participate in periodic reviews by the Health District (p = 0.05) negatively influenced the adherence to the policies.

**Conclusion:**

The low adherence of private health providers to the national guidelines for the management of uncomplicated malaria raises questions about the importance of exercising more control over health providers activities.

## Introduction

En 2015, il a été estimé que près de 214 millions de nouveaux cas de paludisme sont apparus dans le monde ayant entrainé près de 438.000 décès [1, 2]. Cette charge de morbidité et de mortalité est principalement supportée par les pays africains avec 88% des nouveaux cas et 90% de l’ensemble des décès [2]. A Madagascar, le taux d’incidence du paludisme sur l’ensemble de la population est passé de 90 pour mille en 2000 à moins de 10 pour mille en 2010. La mortalité hospitalière a également connu une réduction de plus de 70% [3]. Dans les formations sanitaires de niveau primaire, cette tendance à la baisse dans les consultations externes a été aussi constatée [4, 5]. Cependant, le paludisme constitue encore un problème majeur de santé publique car elle est la première cause de mortalité hospitalière au niveau des hôpitaux de deuxième référence atteignant 21,6% chez les moins de 5 ans [5]. La prévalence du paludisme est estimée à 9% selon une enquête réalisée au niveau national en 2013. Entre l’enquête sur les Indicateurs du Paludisme (EIPM) réalisée en 2011 et en 2013, dans le faciès tropical où la transmission dure plus de 6 mois pendant la saison de pluie, la prévalence est passée de 3% à 12% [6]. Devant cette situation, la politique nationale stratégique 2013-2017 a lancé le défi de l’interruption de sa transmission en passant par la phase de contrôle jusqu’à son élimination [7]. L’adhérence des prestataires de soins aux politiques et directives nationales de lutte contre le paludisme garantit leur succès. Parmi les stratégies utilisées pour atteindre ces objectifs figure l’harmonisation de la prise en charge des cas de paludisme simple par l’utilisation des combinaisons thérapeutiques antipaludiques à base d’artémisinine (ACT) [1]. A Madagascar, un cas de paludisme simple confirmé par le Test de Diagnostic Rapide pour le paludisme (TDR-palu) doit recevoir de l’Artésunate-Amodiaquine par voie orale. Au niveau du secteur privé, l’utilisation des TDR-palu a commencé en 2007 [7]. Cependant, il a été constaté que le secteur privé s’approprie moins de cette politique comparativement au secteur public. Dans une étude de surveillance dans 18 pays, de 2013 à 2015, la proportion d’enfants testés au TDR dans le secteur public est de 53% contre 36% dans le secteur privé formel et de 6% dans le secteur privé informel [1]. Dans le rapport de l’OMS 2015, l’utilisation de TDR devant un enfant fébrile est moins fréquente au niveau du secteur privé par rapport au secteur public [2]. Cette étude se propose: (i) d’identifier les connaissances et attitudes des professionnels de la santé du secteur privé vis-à-vis des directives de prise en charge du paludisme simple; (ii) de déterminer les facteurs influençant l’adhérence des professionnels de santé à l´utilisation du TDR-palu et à la prescription de l’ACT en cas de paludisme simple.

## Méthodes

Une approche évaluative transversale a été conduite en combinant une enquête quantitative et qualitative. Elle a été réalisée dans les Formations sanitaires privés (FSP) se trouvant dans les quatre zones correspondant aux quatre faciès épidémiologiques du paludisme à Madagascar. Dans le faciès équatorial (côte est), la transmission est forte et pérenne. Dans le faciès tropical (côte ouest), cette transmission est saisonnière sur une période de plus de 6 mois correspondant à la saison de pluie. La transmission dans le faciès subdésertique (partie sud) se fait de manière épisodique, instable et courte. Le faciès hauts plateaux et les marges (centres) sont caractérisés par des épisodes épidémiques. Les périodes étudiées concernent les mois de septembre et octobre 2017. Les FSP correspondent aux établissements de soins, hospitaliers ou non hospitaliers, non rattachés à l’Etat, disposant des structures et des ressources permettant de faire des activités curatives, préventives et promotionnelles dans le domaine de la santé [8]. L’adhérence des prestataires de soins des FSP aux recommandations et directives stipulées dans les documents de référence en matière de lutte contre le paludisme a été évaluée notamment à travers les réponses aux questions relatives à leurs connaissances des directives nationales, à leurs attitudes vis-à-vis de ces directives puis par l’observation de leurs pratiques lors de la prise en charge des patients. Un questionnaire individuel pré-testé a été administré auprès des prestataires. Sont appelés « prestataires de soins », les médecins ou paramédicaux qui ont participé au diagnostic et au traitement des cas de paludisme simple venus consulter dans les FSP. Les registres des consultations des établissements de santé ont été également analysés. L’analyse de l’étude qualitative a fait intervenir l’analyse thématique. Le logiciel Epi Info 3.5.3 a été utilisé pour les analyses quantitatives.

**Figure 1:**
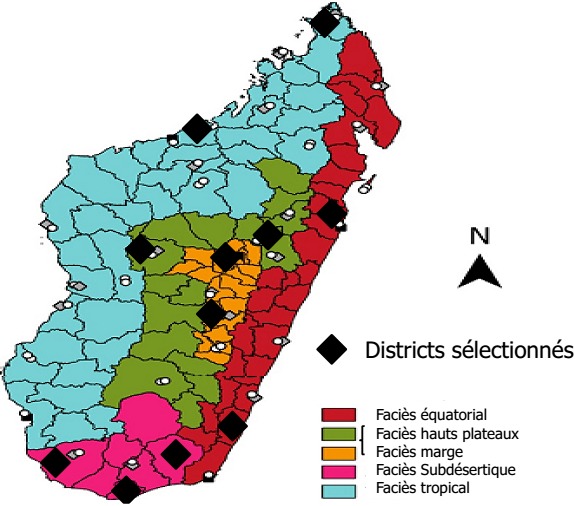
Répartition des faciès épidémiologiques du paludisme à Madagascar avec les districts sélectionnés (source: Direction de Lutte contre le Paludisme)

Un échantillonnage raisonné non probabiliste des districts selon leur profil épidémiologique a permis de sélectionner 11 districts parmi 114 districts sanitaires existant à Madagascar (Figure 1). Au total, 4 districts au niveau des faciès des hauts plateaux et marge (Ambatondrazaka, Antananarivo renivohitra, Fianarantsoa I, Tsiroanomandidy), 2 au niveau du faciès équatorial (Vaingaindrano, Atsinanana I, Atsimo Atsinanana), 2 faciès tropical (Mahajanga I, Antsiranana I) et 3 pour le faciès subdésertique (Tsihombe, Amboasary Atsimo, Tuléar I). Au total, 8 formations sanitaires hospitalières et 31 non hospitalières ont été visitées. Des interviews et observations ont été réalisées auprès de 43 prestataires de soins.

L’analyse statistique a été effectuée sur le logiciel Epi-Info 3.5.3. La comparaison des fréquences relatives a utilisé le test de Chi2. Pour les faibles effectifs, la correction de Yates et de Fischer a été procédée. L’analyse a été réalisée en mode univarié en tenant compte de la taille de l’échantillon. Le seuil de significativité retenu dans cette étude est de p < 0,05. La recherche a démarré après l’obtention de l’approbation du comité d’éthique spécifiée dans sa décision n°04/2015-CEER/INSPC du 24 juillet 2015. La confidentialité, l’anonymat ont été respectés tout au long de l’investigation avec un consentement volontaire et éclairé de chaque participant.

## Résultats

### Concernant les connaissances

Pour les directives nationales, la politique nationale de lutte contre le paludisme et le manuel de prise en charge du paludisme sont disponibles auprès des établissements dans respectivement 30,7% (12/39) et 41,1% (16/39). En réalité, 20,9% (9/43) des prestataires ont lu la politique nationale et 16,3% (7/43) le manuel de prise en charge. Les 97,7% (42/43) des prestataires enquêtés affirment ne pas connaître le faciès épidémiologique du paludisme de la zone où s’implante la FSP. Pour les moyens de diagnostic du paludisme simple, 95,4% des prestataires ont cité le TDR comme le moyen de diagnostic du paludisme simple. Pour les moyens thérapeutiques du paludisme simple, 83,7% des prestataires ont cité l’ACT comme médicament de choix pour traiter le paludisme simple. Il y a une infirmière qui a mentionné la chloroquine, 8 personnes (18,6%) qui ont cité la quinine en comprimé et 9 prestataires issus de FSP non hospitalières (20,9%) qui pensent traiter le paludisme simple avec la quinine injectable.

### Attitudes et pratiques vis-à-vis des directives nationales

#### Avis et utilisation des TDR

Plus de la moitié (55,6%) des prestataires ont évoqué leur doute sur les résultats des TDR. Les raisons avancées par rapport à ces doutes sont nombreuses: l’existence de faux négatifs ou de faux positifs qui aurait été vérifiée après contrôle par la goutte épaisse; des tests négatifs alors que les signes cliniques et l’anamnèse orientent vers un diagnostic probable de paludisme; l’existence de cas à TDR négatifs et qui pourtant ont été guéris par un traitement antipaludique et une possible erreur de fabrication car des fois, il n’y a aucun résultat positif pour tout le lot d’une boîte. Les prestataires favorables aux TDR ont affirmé que les TDR sont faciles à utiliser, accessibles à tout le monde, rapides, pas chers, et permettent d’établir rapidement le diagnostic du paludisme et d’éviter ainsi la prescription abusive d´antipaludéens.

Seul le quart (25,6%) des prestataires dispose de TDR dans leur bureau. Cependant, plus de 8 prestataires sur 10 (83,7%) affirment avoir déjà proposé de réaliser un TDR à leurs patients. Les circonstances ayant motivé la réalisation des TDR sont principalement la fièvre, le constat de signes cliniques en faveur du paludisme, la notion de séjour en zone d’endémie et la persistance d’un état fébrile. Les raisons avancées par ceux qui n’en ont pas proposé à leurs malades sont l’insuffisance de temps, l’absence de confiance en la fiabilité des TDR et l’absence de TDR obligeant le prestataire à référer le patient à une formation sanitaire publique. C’est dans les faciès subdésertiques et hautes terres qu’a été observée la plus forte proportion de prestataires qui n’ont pas proposé des TDR à leurs patients (66,7%). Le Tableau 1 montre la relation entre certaines caractéristiques et le fait de proposer des TDR aux patients fébriles.

**Tableau 1:** Répartition des prestataires de soins selon quelques caractéristiques individuelles et institutionnelles et selon la proposition de TDR aux patients

Caractéristiques	Proposition de TDR aux patients fébriles				Total		P
	Oui		Non				
	Nb	%	Nb	%	Nb	%	
**Type de FSP**							
Hospitalier	12	100	0	0	12	100	0,08
Non hospitalier	24	77,4	7	22,6	31	100	
Lecture de la politique							
Oui	8	88,9	1	11,1	9	100	0,54
Non	28	82,4	6	17,6	34	100	
**Année de sortie des Institutions de formation**							
Avant 2000	20	74,1	7	25,9	27	100	0,03
Après 2000	16	100	0	0	16	100	
Ancienneté au poste							
Moins de 5 ans	13	100	0	0	13	100	0,01
5 à 10 ans	10	100	0	0	10	100	
Plus de 10 ans	13	65	7	35	20	100	
**Formation continue en paludisme**							
Oui	23	79,3	6	20,7	29	100	0,25
Non	13	92,9	1	7,1	14	100	
Ensemble	36	83,7	7	16,3	43	100	

#### Avis et utilisation des ACT

Certains prestataires de soins sont réticents à l’utilisation des ACT qu’ils trouvent difficiles à utiliser dans certains contextes et pour certaines raisons. Les plus grandes difficultés qu’ils ont rapportées sont: le coût exorbitant du produit surtout celui du Coartem^®^; les effets indésirables de l’ACT et les intolérances au médicament, la difficulté d’administration surtout quand le malade présente des vomissements; la non-disponibilité de l’Asaq; la préférence des malades pour les médicaments injectables et leur réticence aux comprimés; la difficulté pour les malades à terminer le traitement. En effet, l’interview des administrateurs des FSP a fait ressortir que 29 FSP sur les 39 FSP (74,4%) ont déclaré avoir disposé d’ACT dans leur stock de médicaments. La plupart des prestataires privés (83,7%) prescrivent l´ACT pour le traitement de paludisme simple même si l’ACT n’est pas disponible dans le FSP.

### Adhérence à la politique de prise en charge du paludisme simple

***Prescription d’antipaludéen devant un résultat de TDR négatif:*** le Tableau 2 fait ressortir que plus du tiers des prestataires (38,2%) prescrit de l’antipaludéen même devant un TDR négatif. Les prestataires qui ont des doutes sur la fiabilité des TDR sont plus nombreux à prescrire des antipaludéens malgré la négativité du TDR par rapport à ceux qui ont pleine confiance aux TDR (p = 0,03).

**Tableau 2:** Répartition des prestataires de soins des FSP selon la prescription d’antipaludéen devant un TDR négatif et selon l’existence de doutes sur la fiabilité des TDR

Caractéristiques	Prescription d'antipaludéen devant un TDR négatif				Total		P
	Oui		Non				
	Nb	%	Nb	%	Nb	%	
**Doutes sur la fiabilité des TDR**							
Oui	10	55,6	8	44,4	18	100	**0,03**
Non	3	18,8	13	81,3	16	100	
Ensemble	13	38,2	21	61,8	34*	100	

* Ont été exclus de l’analyse: 7 prestataires qui n’utilisent pas de TDR et n’en disposent ni dans leurs bureaux ni dans leur centre et 2 qui n’ont pas émis d’avis

***La non-prescription d’ACT devant un résultat de TDR positif:*** le Tableau 3 montre que la moitié des prestataires (50%) déclare avoir prescrit d’autres médicaments que l’ACT devant un TDR positif. Apparemment, la non-prescription d’ACT devant un TDR positif est plus fréquente dans les FSP de type non hospitalier (56% contre 45,5%; NS), chez les paramédicaux (66,7% contre 50%; NS) et chez les diplômés avant l’année 2000 (57% contre 46,7%; NS). Les prestataires qui ne prescrivent pas de l’ACT devant un résultat positif du TDR sont plutôt: ceux qui n’ont lu ni l’exemplaire de la politique nationale de lutte contre le paludisme (53,6% contre 50%; NS), ni le manuel de prise en charge du paludisme (53,3% contre 50%; NS); ceux qui n’ont reçu aucune formation continue en paludisme (58,3% contre 50%; NS) et ceux qui n’ont pas été supervisés ni en interne, ni par une entité externe (60% contre 36,4%; NS). Par contre, les résultats ont clairement démontré que la non-participation aux revues périodiques du district sanitaire favorise significativement la non-prescription d’ACT devant un TDR positif (avec 66,5% contre 33,3%; p = 0,05).

**Tableau 3:** Répartition des prestataires de soins des FSP selon la non-prescription d'ACT devant un TDR positif et selon l’existence de doutes sur la fiabilité des TDR

Caractéristiques	Non-prescription d'ACT devant un TDR positif				Total		P
	Oui		Non				
	Nb	%	Nb	%	Nb	%	
**Doutes sur la fiabilité des TDR**							
Oui	10	55,6	8	44,4	18	100	**0,49**
Non	7	43,8	9	56,3	16	100	
Total	17	50	17	50	34*	100	

### La prescription d’autres molécules en première intention devant un cas de paludisme simple

Les molécules auxquelles les prestataires ont recours en cas de non-prescription d’ACT sont principalement: la quinine sous forme comprimés ou injectables, la sulfadoxine-pyrimethamine. Cette non prescription des ACT relève de plusieurs raisons: l’état du malade ne permet pas d’administrer des médicaments par voie orale (vomissement); l’ACT n’est pas disponible au centre; le malade ne peut pas acheter l’ACT; le malade lui-même réclame un produit injectable étant convaincu que c’est le plus efficace; certains malades ne tolèrent pas l’ACT.

## Discussion

Cette étude a montré que la connaissance de l’intérêt à réaliser un TDR devant un patient fébrile et à prescrire l’ACT en cas de résultat positif du TDR demeure élevée. Cependant, en pratique, il a retrouvé que plus du tiers des prestataires (38,2%) prescrit de l’antipaludéen même devant un TDR négatif. En plus, l’ACT n’est pas prescrit systématiquement pour la moitié des prestataires (50%) en cas de TDR positif. Les doutes sur les résultats du TDR sont élevés (55,5%). Cette situation influence ainsi l’adhérence aux directives. Malgré la faible taille de l’échantillon, la représentativité des districts a été adaptée aux faciès épidémiologiques du paludisme à Madagascar. Les réponses des prestataires à certaines questions de perception peuvent induire des biais d’information du fait de leur subjectivité. Cependant, l’observation directe de leurs pratiques devant la prise en charge d’un cas de fièvre a permis de vérifier la qualité de la prise en charge. Pour éviter des changements de comportement ou de pratique qui pourraient survenir devant la présence de l’investigateur, l’observation a consisté à comparer les motifs de consultation des patients fébriles et les prescriptions du professionnel de la santé.

### Connaissances et attitudes

Il est important pour le prestataire de connaître la politique en vigueur afin de s’y conformer. Cette connaissance doit aussi s’intéresser au faciès épidémiologique afin de bien adapter le schéma thérapeutique. En effet, les directives thérapeutiques sont différentes selon les faciès [1]. Or, les documents de référence (Politique Nationale de lutte contre le paludisme, Manuel de prise en charge du paludisme) ne sont pas disponibles au niveau de bon nombre d’acteurs dans le secteur privé. Et quand ils sont disponibles dans un établissement, son partage en interne est encore à améliorer [8]. Pour le TDR, la démarche diagnostique commence par l’interrogatoire et l’examen clinique. Mais la politique nationale exige le recours au TDR pour établir le diagnostic du paludisme [7]. D’autres examens biologiques permettent aussi d’établir le diagnostic pour ne citer que la goutte épaisse mais dans un contexte limité, l’usage des TDR est de plus en plus recommandé dans différents contextes. Dans d’autres pays africains, l’utilisation des TDR est même attribuée aux agents communautaires formés et aux pharmacies détaillants [9]. Dans cette étude, la faible disponibilité des TDR dans les bureaux contraste avec l’affirmation des prestataires disant avoir déjà réalisé des TDR. Cette faible disponibilité est d’ailleurs retrouvée dans d’autres études comme celle réalisée au Nigéria où elle est de moins de 20% des FSP contre 82% dans les formations sanitaires publiques [10]. Le fait que certains prestataires de soins parlent d’autres molécules (quinine injectable et chloroquine) que l’ACT comme médicament de première intention en cas de paludisme simple reflète un manque de connaissance. Cette situation a été aussi décrite en 2011 où 6% de ces enfants ont bénéficié de l’ACT dont l’Asaq est le plus utilisé (4%), quinine (3%) et chloroquine (2%) [6]. Pour la quinine comprimé, elle peut être prescrite pour traiter le paludisme simple chez une femme enceinte. Par contre, recourir directement à la quinine injectable devant un paludisme simple va à l’encontre des directives nationales. Dans le milieu privé, la promotion des médicaments par les distributeurs et commerciales influence la prescription [10].

### Pratiques des prestataires

L’adhésion des prestataires aux directives nationales s’apprécie à travers leurs pratiques en matière de démarche diagnostique et de traitement des cas de paludisme simple. La disponibilité des TDR dans les FSP de type hospitalier explique leur utilisation plus élevée. D’ailleurs, les hôpitaux privés se trouvent dans les grandes villes facilement accessibles. Les médecins sont plus réticents car ils se fient à leur expérience clinique [9]. Paradoxalement, la formation continue en paludisme, qui normalement aurait dû améliorer les pratiques des prestataires [11] a entraîné l’effet inverse. Par contre, les prestataires ayant fini leurs études après l’année 2000 et ceux qui ont une ancienneté de moins de 10 ans ont, pour la totalité, proposé le TDR à leurs patients fébriles. Ils sont plus adhérents car ils ne sont pas encore dans la routine et la formation initiale a permis d’éviter les anciennes pratiques.

### Adhérence aux directives

Devant un résultat positif du TDR, la politique nationale recommande d’utiliser l’ACT pour traiter la maladie. Mais tel n’est pas toujours le cas ainsi que l’illustrent les résultats des analyses des données. Si le TDR est négatif, les prestataires ont toujours tendance à faire passer leur impression clinique. D’ailleurs, dans certains pays africains, les médecins sont les plus réticents aux résultats négatifs des TDR [9]. L’analyse de plusieurs études déjà réalisées sur la proportion de prestataires prescrivant de l’ACT devant un TDR négatif a montré une fourchette entre 2%-83% dont la majorité est inférieure à 20% [11]. La proportion retrouvée dans cette étude semble donc être plus élevée mais à un degré moindre comparativement à une étude menée au Nigéria [12]. Une étude réalisée au Kenya a montré que la confiance des prestataires aux directives influence sur la confiance des patients et leur adhérence thérapeutique. En effet, ceux qui ont reçu de l’ACT malgré un TDR négatif (49%) disent ne pas être confiants au traitement comparativement à ceux traités par ACT (89%) lorsque le TDR est positif ou sans TDR [13]. Il a été démontré que la disponibilité des TDR et des ACT dans les formations sanitaires privées améliore significativement la gestion des cas de paludisme et la confiance des patients [14, 15]. A côté de cette notion de disponibilité se trouve la notion d’accessibilité. En effet, Il faut noter que l´utilisation des ACT est freinée du fait de leur accessibilité financière surtout pour les prix non subventionnés [16]. En effet, les ACT sont 10 à 20 fois plus chers que les autres antipaludéens dans les pharmacies privées. Plus les FSP se trouvent dans des zones reculées, plus le coût des ACT augmente d’après l’évaluation des ACT subventionnés (ACTm) [17]. Les doutes sur les résultats des TDR et l’utilisation d’autres molécules que les ACT dans le paludisme simple sont liés à l’insuffisance des activités d’encadrement et de renforcement des compétences des acteurs dans le secteur privé (formation, participation aux revues périodiques du district, supervisions). D’ailleurs, des études ont confirmé cette situation où une formation plus poussée, un prix abordable des TDR et une meilleure supervision constitueraient des facteurs associés à une meilleure adhérence des prestataires [11]. D’ailleurs, une étude réalisée en Ethiopie a montré qu’un meilleur accompagnement du secteur privé a amélioré significativement l’adhérence à la politique passant de 47,8% à 95,7% [18].

## Conclusion

Cette étude a montré qu’une grande partie des prestataires de soins travaillant au niveau du secteur privé à Madagascar connaissent les grandes lignes des directives sur la prise en charge des cas de paludisme simple. Toutefois, l’adhérence à ces directives reste faible à cause des doutes sur les résultats des TDR. Cette recherche a mis en exergue la nécessité d’encadrer davantage le secteur privé dans le respect des directives de la politique de prise en charge du paludisme. Sans vouloir extrapoler à tout le secteur privé, les résultats obtenus permettent de réorienter les actions à entreprendre auprès de ce secteur.

### Etat des connaissances actuelles sur le sujet

- Le traitement du paludisme simple doit être précédé d’un test de diagnostic rapide avant l’instauration d’une combinaison thérapeutique à base d’artémisinine;
- La lutte contre le paludisme implique l’adhésion aussi bien du secteur public et du secteur privé- Le professionnel de santé travaillant dans le secteur public est souvent bien encadré;
- Cependant, l’harmonisation des directives et le suivi du secteur privé restent méconnus.

### Contribution de notre étude à la connaissance

- Evaluation des connaissances, attitudes et pratiques des prestataires de soins au niveau du secteur privé;
- Identification des facteurs associés à l’adhérence aux politiques de prises en charge du paludisme simple;
- Cette étude apporte les éléments à tenir en compte pour améliorer l’implication du secteur privé dans la lutte contre le paludisme.

## Conflits d’intérêts

Les auteurs ne déclarent aucun conflit d’intérêts.
